# Melatonin Absence Leads to Long-Term Leptin Resistance and Overweight in Rats

**DOI:** 10.3389/fendo.2018.00122

**Published:** 2018-03-27

**Authors:** Daniella Buonfiglio, Rafaela Parthimos, Rosana Dantas, Raysa Cerqueira Silva, Guilherme Gomes, Jéssica Andrade-Silva, Angela Ramos-Lobo, Fernanda Gaspar Amaral, Raphael Matos, José Sinésio, Lívia Clemente Motta-Teixeira, José Donato, Russel J. Reiter, José Cipolla-Neto

**Affiliations:** ^1^Department of Physiology and Biophysics, Institute of Biomedical Sciences-I, University of São Paulo (USP), São Paulo, Brazil; ^2^Department of Physics and Interdisciplinary Science (FCI), São Carlos Institute of Physics (IFSC), University of São Paulo (USP), São Paulo, Brazil; ^3^Department of Physiology, Universidade Federal de São Paulo – Unifesp, São Paulo, Brazil; ^4^Department of Cellular and Structural Biology, UT Health Science Center, San Antonio, TX, United States

**Keywords:** melatonin, feeding behavior, pinealectomy, leptin resistance, signal-transducer and activator of transcription 3, brown adipose tissue, thermogenesis, overweight

## Abstract

Melatonin (Mel), a molecule that conveys photoperiodic information to the organisms, is also involved in the regulation of energy homeostasis. Mechanisms of action of Mel in the energy balance remain unclear; herein we investigated how Mel regulates energy intake and expenditure to promote a proper energy balance. Male Wistar rats were assigned to control, control + Mel, pinealectomized (PINX) and PINX + Mel groups. To restore a 24-h rhythm, Mel (1 mg/kg) was added to the drinking water exclusively during the dark phase for 13 weeks. After this treatment period, rats were subjected to a 24-h fasting test, an acute leptin responsiveness test and cold challenge. Mel treatment reduced food intake, body weight, and adiposity. When challenged to 24-h fasting, Mel-treated rats also showed reduced hyperphagia when the food was replaced. Remarkably, PINX rats exhibited leptin resistance; this was likely related to the capacity of leptin to affect body weight, food intake, and hypothalamic signal-transducer and activator of transcription 3 phosphorylation, all of which were reduced. Mel treatment restored leptin sensitivity in PINX rats. An increased hypothalamic expression of agouti-related peptide (Agrp), neuropeptide Y, and Orexin was observed in the PINX group while Mel treatment reduced the expression of Agrp and Orexin. In addition, PINX rats presented lower UCP1 protein levels in the brown adipose tissue and required higher tail vasoconstriction to get a proper thermogenic response to cold challenge. Our findings reveal a previously unrecognized interaction of Mel and leptin in the hypothalamus to regulate the energy balance. These findings may help to explain the high incidence of metabolic diseases in individuals exposed to light at night.

## Introduction

Body weight is the final result of an energy balance. The complex mechanism that controls food intake relies on the central nervous system and involves the hypothalamus, striatum, brain stem, and other structures. The hypothalamic food intake regulatory system is composed of orexigenic and anorexigenic neurons and by their post-synaptic targets in order to induce food intake or satiety. Many hormones and neurotransmitters affect this neural system causing changes in feeding behavior (1–3). Energy expenditure, on the other hand, depends on several physiological and behavioral phenomena including the activation of the brown adipose tissue (BAT) (4). Besides energy intake and energy expenditure, energy storage is also pivotal for the energy balance and is regulated by several neural and endocrine factors such as insulin, leptin, glucocorticoids, and growth hormone (5).

Melatonin (Mel), the major circadian mediator of photoperiodic information to the central nervous system, promotes energy homeostasis regulating energy balance (6). Extensive data in different species show that Mel is involved in the regulation of the three major steps in energy balance: food intake, energy storage, and energy expenditure (7, 8). As a consequence, administration of Mel in drinking water or liquid diet leads to a reduction of body weight and abdominal fat in rodent models of hyperadiposity. In rats, Mel supplementation can reduce body weight and fat mass independently of food intake reduction (9–13). In the zebrafish *Danio rerio*, Mel inhibits orexigenic and stimulates anorexigenic signals in the CNS, leading to a reduction in food intake (14). Data from our group showed that rhythmic Mel treatment *in vitro* synchronizes the white adipocyte metabolic and hormonal functions, including leptin secretion (15). More recently, Mel was shown to be a powerful synchronizing agent for leptin secretion in *Syrian hamsters* (16).

One of the main actions of Mel is to participate in the circadian organization of metabolic functions and their association to the circadian behavioral cycles regulating food intake and energy expenditure (6). Moreover, Mel drives the daily rhythmicity of plasma leptin and modulates the glycemic homeostasis according to the photoperiod-dependent metabolic state (16). Mel is essential for coupling metabolic functions to the light–dark cycle ensuring metabolic homeostasis. The uncoupling of these functions is related to metabolic syndrome and obesity.

Leptin is an important anorexigenic signal. It is produced and secreted mainly by adipocytes, in proportion to adipose mass (17). Most of the well-studied biological roles of leptin are CNS-mediated through the activation of its membrane receptor (LepR). LepR belongs to the cytokine receptor superfamily and signals *via* a number of downstream pathways, including the Janus kinase 2 and the signal-transducer and activator of transcription 3 (STAT3) pathway. Phosphorylated STAT3 acts as a transcription factor that regulates the expression of target genes, including suppressor of cytokine signaling 3, a critical negative-feedback regulator of LepR signaling (18, 19). In the hypothalamus, the arcuate nucleus (ARH) is a fundamental site for leptin’s action. ARH is composed by different neuronal populations that express LepR, such as proopiomelanocortin (POMC) positive neurons that are stimulated by leptin. POMC can be cleaved in several peptides, including the α-melanocyte-stimulating hormone (α-MSH) that has anorexigenic effects. In ARH, there is another population of neurons that co-express neuropeptide Y (NPY) and the agouti-related protein (AgRP). The NPY/AgRP neurons present orexigenic effects and are inhibited by leptin. Leptin regulates energy stocks through its action in the CNS because of its direct access to neurons responsible for the feeding behavior and other aspects of energy metabolism (5, 20). Other important hormones for the regulation of energy balance are the anorexigenic cortico releasing hormone (CRH) released in the paraventricular nucleus which are suggested to mediate leptin’s effects on feeding and adiposity (21); and the orexigenic melanin-concentrating hormone (MCH), which acts increasing AgRP/NPY and decreasing POMC/CART (22).

Leptin resistance and consequently, hyperleptinemia, seems to be one of the major causes of obesity. However, the mechanism through which leptin resistance is triggered has yet to be identified (23, 24).

Melatonin is critically involved in leptin synthesis and release by white adipocytes (15, 25, 26) and its absence is related to metabolic syndrome, diabetes, and increased body weight. Moreover, the therapeutic replacement or supplementation of Mel reduces body weight by reducing adiposity, modulates energy expenditure, and possibly food intake (6). Our hypothesis is that Mel or its absence modulate energy metabolism by acting in the leptin pathway. Notwithstanding this functional correlation, the molecular and signaling interactions between Mel and leptin actions remain unclear. Therefore, it becomes essential to determine how leptin signaling and behavioral effects are impacted by the absence of Mel and its replacement. Understanding the relationship among Mel, leptin and energy metabolism is critical to improving strategies to limit obesity, especially in individuals exposed to environmental factors and circadian disturbances that affect Mel secretion.

## Materials and Methods

### Animals and General Procedures

Male Wistar rats were maintained under a 12-h light/12-h dark cycle at the animal facility room of the Neurobiology Lab, Department of Physiology and Biophysics, University of São Paulo. All the experimental procedures were approved by the Ethics Committee on Animal Use of the Institute of Biomedical Sciences of the University of São Paulo, under the number 86/2016.

Animals were assigned to four groups: control, intact animals; control + Mel, intact animals that were supplemented with Mel; pinealectomized (PINX) animals; and PINX + Mel, PINX animals that were treated with Mel since the day of surgery. Mel (1 mg/kg of body weight) was added to the drinking water exclusively during the dark phase and the concentration in the drinking water solution was corrected on a daily basis using the previous night ingested volume and the body weight of each animal. To determine changes in energy balance, food intake, and body weight were measured three times a week. All groups were fed with regular chow; 2.99 kcal/g; 9.4% kcal derived from fat; Quimtia, Brazil. Table 1 summarizes the experimental setup.

**Table 1:** Experimental setup.

	Groups	Experimental procedures				Euthanasia and analysis
Experiment 1, *n* = 10/group	CTLPINXCTL + MelPINX + Mel(13 weeks of melatonin treatment)	Pinealectomy surgery at 2 months of age in PINX and PINX + Mel groups	Body weight and food intake 3× a week for 13 weeks	At 11 weeks: 24 h fasting test	At 12 weeks: acute leptin sensitivity test	At 13 weeks: PBS (*n* = 5) or leptin (*n* = 5) injection for WB of hypothalami (pSTAT3) and BAT(UCPl); adiposity
Experiment 2, *n* = 10/group	CTLPINXPINX + Mel(13 weeks of melatonin treatment)	Body weight and food intake 3× a week for 13 weeks				At 13 weeks: hypothalami collection to perform mRNA analysis
Experiment 3, *n* = 10/group	CTLPINX	Pinealectomy surgery at 2 months of age in PINX and PINX + Mel groups	Body weight and food intake 3x a week for 36 weeks		At 34 weeks: Cold Challenge	At 36 weeks
Experiment 4, *n* = 5/group	CTLPINX	ICV cannula implantation and pinealectomy (in PINX group) surgeries at 2 months of age	7 days to measure basal (no injection) food intake		Ringer (BuL) or melatonin (90 ng in SuL) injection: 30 days to measure food intake	At 14 weeks

### Pinealectomy

Eight-week-old rats were anesthetized with intraperitoneal injection of ketamine and xilazin (0.15 ml/100 g body weight) and fixed in a stereotaxic apparatus. A sagittal opening was made on their scalp, after pushing the skin aside to expose the lambdoid suture, a disk-shaped perforation was made around the lambdoid suture by a circular drill, and a disk-shaped piece of bone was delicately removed. The superficial pineal gland, located below the posterior venous sinus confluence, was removed using a fine forceps. After the pineal gland’s removal, the disk-shaped piece of bone was returned to the skull and the scalp was sutured with cotton threads after a brief hemostasis (27).

### Adiposity and Hormone Levels

Subcutaneous, perigonadal (PE), and retroperitoneal (RP) fat pads were collected and weighed to determine the amount of adipose tissue in each group. Fat mass was normalized by body weight for comparison between groups. Serum was collected from trunk blood for leptin and insulin measurements by MILLIPLEX^®^ Multiplex Assays/Merck.

### Acute Leptin Sensitivity Test

To assess the acute sensitivity to leptin, animals received an intraperitoneal injection of phosphate-buffered saline (PBS) at ZT 10 (2 h before the beginning of dark phase) and their food intake and body weight were recorded 12 and 24 h after injection. The day after, rats received an intraperitoneal injection of rat-recombinant leptin (0.6 mg/kg of body weight; from Dr. A. F. Parlow, National Hormone and Peptide Program, National Institute of Diabetes and Digestive and Kidney Diseases, NIH, USA) and their food intake and body weight were recorded 12 and 24 h after injection. The purpose of using a within-animal control was to minimize interindividual variability in the assessment of the anorexigenic effects of leptin. Therefore, each rat acts as its own control.

To evaluate leptin activation of its intracellular signaling pathway, STAT3 phosphorylation was assessed after an acute leptin stimulus (0.6 mg/kg of body weight) (23, 28, 29). Rats (*n* = 5/group) received an intraperitoneal injection of PBS or leptin and were euthanized 90 min later at ZT4. Hypothalami were collected and stored at −80°C for pSTAT3 western blot analysis.

### 24-h Fasting Response

The 24-h fasting response was assessed by removing food from the cages and re-feeding all animals after 24 h. Body weight and food intake were monitored 6, 12, and 24 h after fasting and 6 and 12 h after re-feeding.

### Intracerebroventricular (ICV) Mel Injection

Ten-week-old male Wistar rats were anesthetized with Xylazin and Ketamine i.p. (0.15 ml/100 g body weight) and placed in a stereotaxic apparatus (David Kopf Instruments, CA, USA). One hole was drilled on the parietal bone (2.0 mm posterior to bregma, midline), the guide cannula was ventrally pushed into the third ventricle (7.5 mm from the skull) and fixed in a vertical position with dental cement (copolymer of acrylic and acrylic self-polymerizing Articles Dental Classic^®^, São Paulo, SP, Brazil) and two lateral screws. In the rats of PINX group, the removal of the pineal gland was performed as previously described, immediately before the guide cannula implantation.

Intracerebroventricular injections were initiated 7 days after surgery. The daily injections started immediately before the light/dark transition and the total volume injected was 5 µl of vehicle (Ringer’s solution: 147 mM NaCl, 4 mM KCl, 1.2 mM CaCl2, and 1.0 mM MgCl2) or 5 µl of Mel (18 ng/µl in vehicle). The solutions were maintained at 37^°^C. All animals were injected daily for 30 days and the 24-h food consumption was evaluated daily. The injection system consisted of a probe (0.28 mm diameter), a tubing adaptor, and polypropylene tubing that was connected to a 10 µl Hamilton syringe. The system was fully filled with either vehicle or Mel solution prior to injection. The injection lasted about 2 min and the injection system allowed free movement for the animals during ICV injection.

### Analysis of Genes Involved in the Hypothalamic Control of Feeding Behavior

To evaluate the expression of genes involved in feeding behavior, hypothalami were collected and submitted to RNA isolation by the guanidine isothiocyanate extraction method using TRIzol^®^ Reagent (Invitrogen, Carlsbad, CA, USA). The purity was assessed by the 260/280 nm ratio and the quantity measured at 260 nm. After that, cDNA was synthesized from 1 µg of total RNA using the ImProm-II (Promega). Real-time PCR analysis of Npy, AgRP, Orexin, Mch, Pomc, Cart, and Crh genes were performed (Primers design, Table 2) by using Power SyBR Green (Applied Biosystems) in the QuantStudio 6 Flex Real-Time PCR System (Thermo Fisher Scientific). The data were analyzed by 2^−ΔΔCT^ and normalized by geometric mean of Gapdh, Rpl, beta-actin, and HDAC.

**Table 2:** Real-time PCR primers.

Gene	GenBank accession number	Primer sequences (5′–3′)	Product size (bp)
*Agrp*	NM_033650.1	F: GCAGAGGTGCTAGATCCACAGAA R: AGGACTCGTGCAGCCTTACAC	70
*β-actin*	NM_031144.2	F: CCCTGGCTCCTAGCACCAT R: GAGCCACAATCCACACAGA	75
*Cartpt*	NM_017110.1	F: CCGAGCCCTGGACATCTACT R: CCGCCTTGGCAGCTCCTT	67
*Crh*	NM_031019.1	F: TGGATCTCACCTTCCACCTTCTG R: CCGATAATCTCCATCAGTTTCCTG	103
*Gapdh*	NM_017008.4	F: GGGCAGCCCAGAACATCAT R: CCGTTCAGCTCTGGGATGAC	76
*Hcrt (orexin)*	NM_013179.2	F: GCGGCCTCAGACTCCT R: AGGGAGAGGCAATCCGGAGAG	70
*Hdac1*	NM_001025409.1	F: TGGTCTCTACCGAAAAATGGAAA R: GTCGTCGCTGTGGTACTTGGT	78
*MCH*	M29712.1	F: ATGCTGGCCTTTTCTTTGTTT R: CTTCTACGTTCCTGATGGACTT	70
*Npy*	NM_012614.2	F: CCGCCCGCCATGATGCTAGGTA R: CCCTCAGCCAGAATGCCCAA	73
*Pomc*	NM_139326.2	F: ATAGACGTGTGGAGCTGGTGC R: GCAAGCCAGCAGGTTGCT	75
*Rpl37a*	NM_001108801	F: CGCTAAGTACACTTGCTCCTTCTG R: GCCACTGTTTTCATGCAGGAAC	93

### Western Blot Analysis

Immediately after collection, the hypothalamus or BAT was homogenized in RIPA buffer (Sigma) containing a cocktail of protease and phosphatase inhibitors (1:100, Sigma), centrifuged (14,000 RPM, 4°C for 20 min) and the supernatants were retained. After determination of total protein concentration (Pierce BCA Protein Assay, Thermo Scientific) 50 µg of total protein was loaded in a 10% SDS-PAGE gel and finally transferred to a nitrocellulose membrane (Bio-Rad). Membranes were blocked with 5% bovine serum albumin and incubated overnight at 4°C using commercially available primary antibodies (1:1,000) to identify pSTAT3^Tyr705^ (Cell Signaling), UCP1 (Santa Cruz), or β-Actin (Sigma). Next, we incubated the membranes for 60 min in 1:20,000 secondary antibody (IRDye 800CW, Li-COR). Proteins were detected by fluorescence, analyzed using the Li-COR Odyssey system (Li-COR), and normalized to β-actin or Ponceau staining (30).

### Thermography—Cold Challenge

To assess the thermogenic responses to a cold challenge, animals were fasted overnight and individually placed in a 4-l glass beaker surrounded by ice at ZT15. The heat response of the BAT (maximum temperature irradiated from the dorsal interscapular region), maximum eye temperature, and temperature of the tail (average of tail temperature) were recorded, at baseline and after the cold challenge, with an infrared camera (Model: Sc6040—Flir Systems) for 60 min each (capture rate: 0.2 frame/s). Each thermography has a 12-bit image (640 × 480 pixels) and was obtained in IR wavelength electromagnetic radiation (8–12 mm), and each pixel with 0.1°C resolution. The temperature of the environment and substrate were also obtained to correlate with the temperature of the body surface. For all measurements, emissivity coefficient in the camera was set to 0.95 as estimated for biological tissues (31).

### Statistical Analyses

Results are presented, as mean ± SEM. Data were assessed for normality and homogeneity of variance by D’Agostino-Pearson omnibus test. *p*-Values were determined according to each experimental design by *t*-test, one-way ANOVA or two-way ANOVA as presented in figures legend. The significance level was *p* < 0.05. Statistical analyses and graphics were performed using GraphPad Prism V.6 software.

## Results

### Mel Reduces Food Intake, Body Weight, and Adiposity

**Figure 1:**
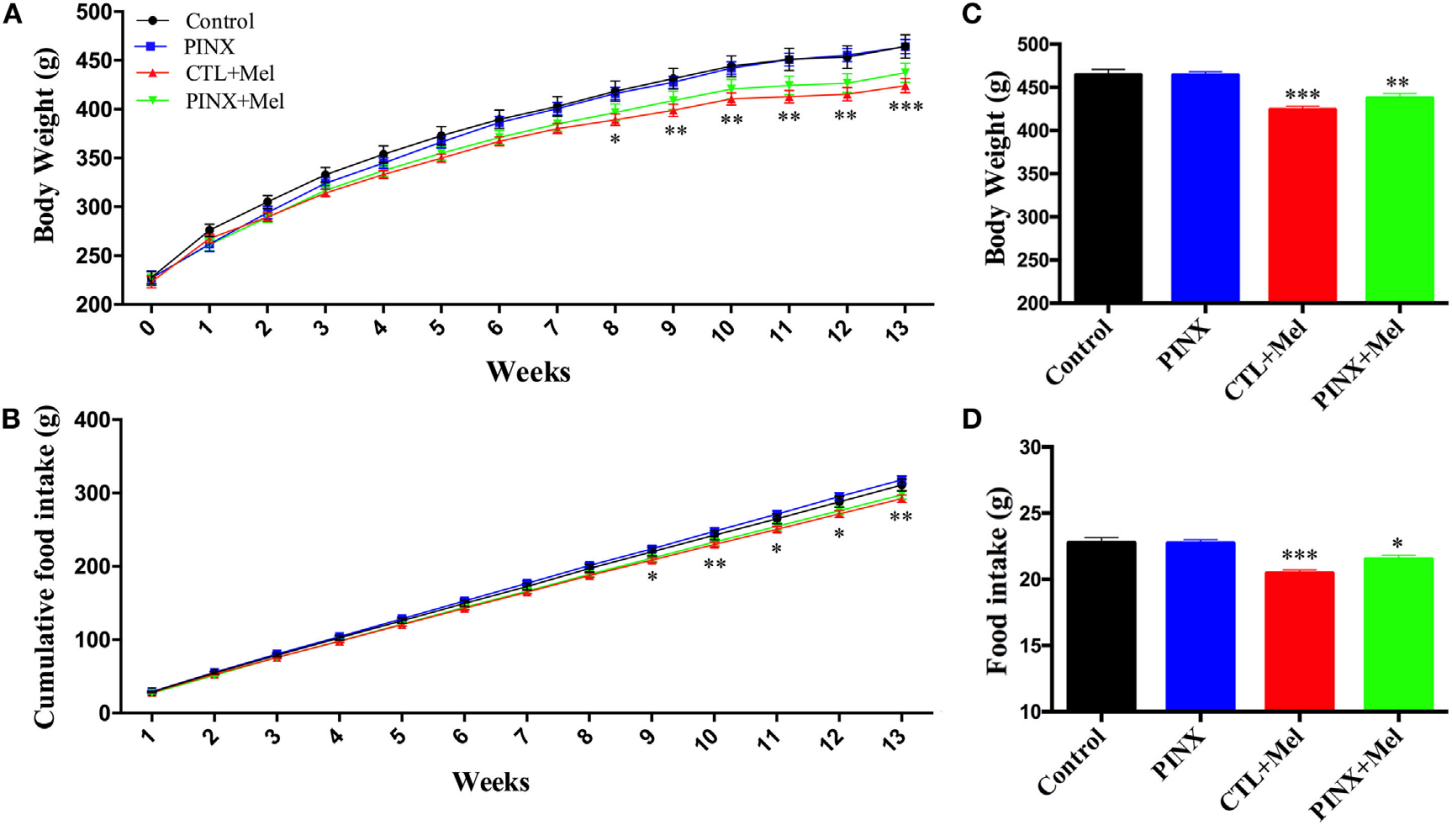
Effect of circadian melatonin (Mel) treatment on body weight and food intake. **(A)** Body weight gain over the course of 13 weeks of Mel treatment. Two-way RM ANOVA followed by Tukey post-test *p < 0.05 CTL vs CTL + MEL; **p < 0.005 MEL-TREATED groups vs control and pinealectomized (PINX); ***p < 0.001 MEL-TREATED groups vs control and PINX. Interaction *p* < 0.0001 *F*(39, 481) = 4.013; weeks *p* < 0.0001 *F*(13, 481) = 1,726; treatment *p* = 0.0387 *F*(37, 481) = 58.96; subjects *p* < 0.0001 *F*(37, 481) = 58.96. **(B)** Food intake throughout 13 weeks of Mel treatment. Two-way RM ANOVA followed by Tukey post-test *p < 0.05 PINX vs CTL + MEL; **p < 0.005 MEL-TREATED groups vs control and PINX. Interaction *p* < 0.0001 *F*(36, 432) = 3.408; weeks *p* < 0.0001 *F*(12, 432) = 8,432; treatment *p* = 0.0534 *F*(3, 36) = 2,807; subjects *p* < 0.0001 *F*(36, 432) = 41.96. **(C)** Daily body weight at the end of Mel treatment protocol. Ordinary one-way ANOVA followed by Tukey post-test, **p < 0.0040 vs control and PINX; ***p < 0.0001 vs control and PINX. Treatment *p* = 0.0001 *F*(3, 119) = 15.11. **(D)** Daily food intake at the end of Mel treatment protocol. Ordinary one-way ANOVA followed by Tukey post-test, *p < 0.0299 vs control and PINX; ***p < 0.0001 vs control and PINX. Treatment *p* = 0.0001 *F*(3, 117) = 15.03. Data are shown as mean ± SEM, *n* = 10/group.

**Figure 2:**
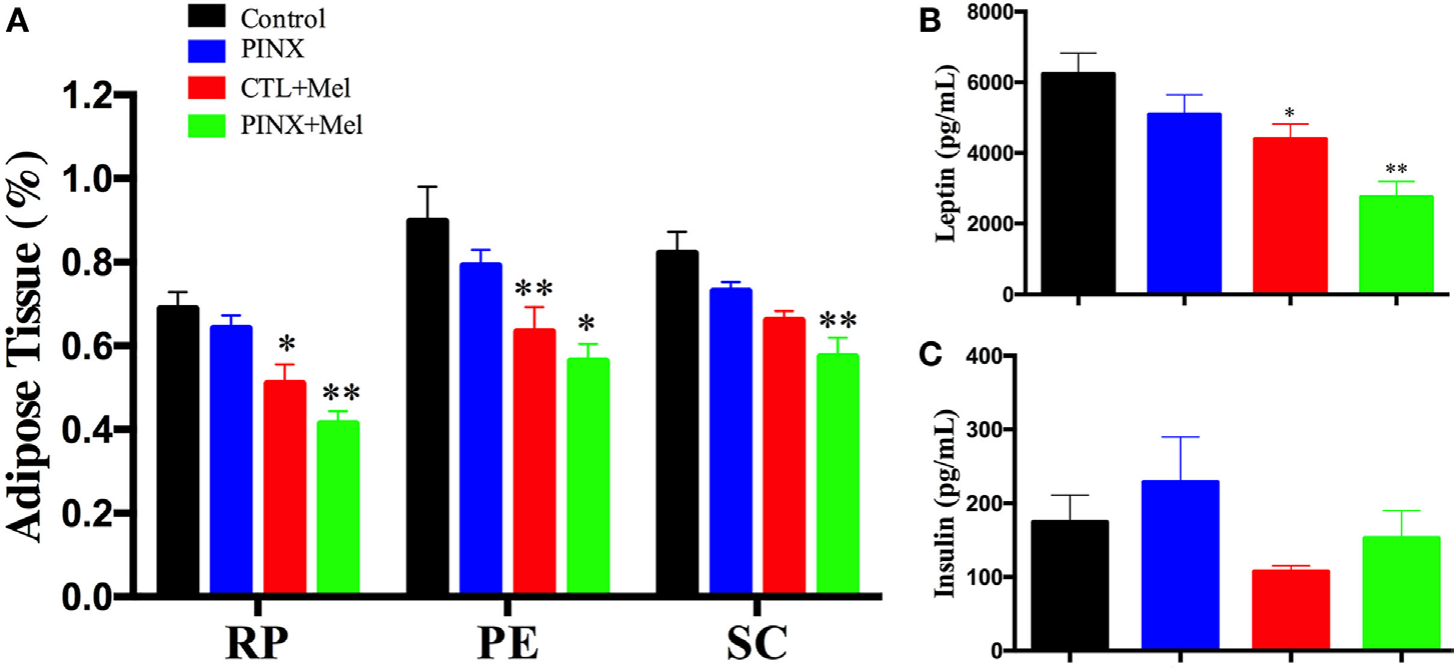
Effect of circadian melatonin (Mel) treatment on rat adiposity, leptin, and insulin serum levels. **(A)** Amount of adipose tissue after Mel treatment in retroperitoneal (RP), perigonadal (PE), and subcutaneous fat pads. Data of fat mass were normalized by body weight. Two-way ANOVA followed by Tukey post-test. RP: *p < 0.05 vs control, **p < 0.001 vs pinealectomized (PINX) and control; PE: **p < 0.001 vs control, *p < 0.01 vs PINX; SC: **p < 0.001 vs control. Interaction *p* = 0.8949 *F*(6, 104) = 0.3727; fat pad factor *p* < 0.0001 *F*(2, 104) = 12.84; treatment *p* < 0.0001 *F*(3, 104) = 28.00. **(B)** Serum leptin levels after Mel treatment. One-way ANOVA followed by Sidak post-test, *p < 0.05 vs control, **p < 0.001 vs PINX. Treatment *p* = 0.0002 *F*(3, 50) = 8.019. **(C)** Serum insulin levels after Mel treatment. No statistical difference was observed between groups. Treatment *p* = 0.0692 *F*(3, 33) = 2.593. Data are shown as mean ± SEM, *n* = 10/group.

The role of Mel in the regulation of energy balance was assessed by analyzing food intake, body weight, and adiposity of control, control + Mel, PINX, and PINX + Mel rats. Treated animals received Mel in the drinking water during the dark phase. Figure 1A shows that after 9 weeks of Mel treatment, body weight gain in the control + Mel and PINX + Mel groups began to fall behind of the control and PINX groups (**p < 0.005 vs control and PINX; ***p < 0.001 vs control and PINX), and after 13 weeks (Figure 1C) the groups treated with Mel had a highly significant lower body weight (***p < 0.0001 vs control and PINX, **p < 0.0040 vs control and PINX). The same can be seen for food intake. The cumulative food intake curves for treated groups were below the respective untreated groups by 9 weeks of Mel treatment (Figure 1B; *p < 0.05 vs PINX and CTL; **p < 0.005 vs PINX and CTL), showing a clear significant effect at the end of the 13 weeks of treatment (Figure 1D; ***p < 0.0001 vs control and PINX, *p < 0.0223 vs control and PINX). No differences were observed in the body weight, food intake, and adiposity of control group compared with PINX group 13 weeks after pinealectomy (Figures 1 and 2). However, Mel treatment reduced adiposity in both control + Mel and PINX + Mel groups in almost all territories of adipose tissue analyzed (Figure 2A; RP: *p < 0.05 vs control, **p < 0.001 vs PINX; PE: **p < 0.001 vs control, *p < 0.01 vs PINX; SC: **p < 0.001 vs control). This result is in agreement with the serum leptin levels that were reduced in Mel-treated groups compared with control and PINX groups (Figure 2B; *p < 0.05 vs control group, **p < 0.01 vs PINX group). No statistical differences were found in serum insulin levels (Figure 2C).

**Figure 3:**
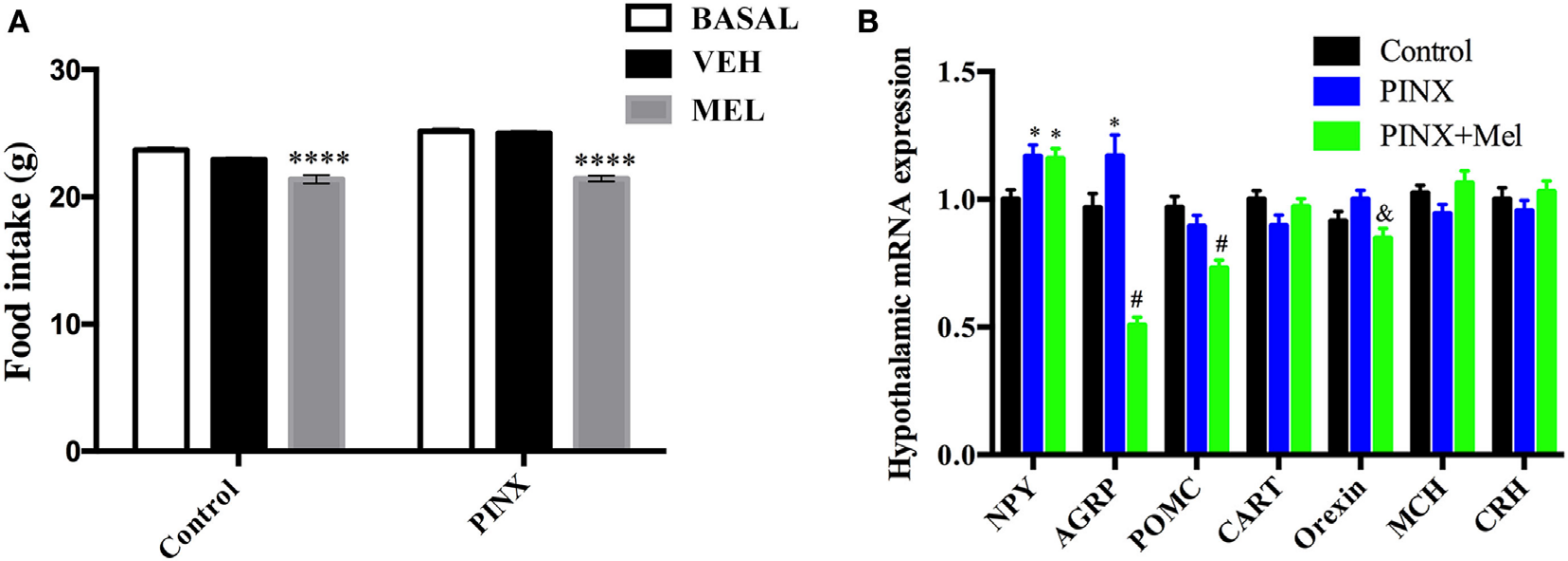
Expression of genes related to food intake and the intracerebroventricular (ICV) melatonin (Mel) effects on food intake. **(A)** Food intake after an ICV Mel injection for 30 days. Two-way ANOVA followed by Tukey post-test, ****p < 0.0001 vs basal and vehicle group. VEH: vehicle group. Interaction *p* < 0.0001 *F*(2, 945) = 10.97; group factor *p* < 0.0001 *F*(1, 945) = 54.90; treatment *p* < 0.0001 *F*(2, 945) = 119.2. Data are shown as mean ± SEM, *n* = 15/group. **(B)** Real-time PCR analysis of hypothalamic *Npy, Agrp, Pomc, Cart, Orexin, Mch*, and *Crh* mRNA expression in Mel presence or absence. Each value represents the target gene expression corrected by the geometric mean of housekeeping genes expression. Two-way ANOVA followed by Tukey post-test, *Npy*: *p < 0.02 vs control group; *Agrp*: *p < 0.005, ^#^*p* < 0.0001 vs control group; *Pomc*: ^#^*p* < 0.001 vs control and pinealectomized (PINX) groups; *Orexin*: ^&^*p* < 0.05 vs PINX. Interaction *p* < 0.0001 *F*(12, 585) = 12.36; gene factor *p* < 0.0001 *F*(6, 585) = 12.22; groups *p* < 0.0001 *F*(2, 585) = 11.70. No statistical differences were observed between groups on *Cart, Mch*, and *Crh* expression. Data are shown as mean ± SEM, *n* = 10/group.

Melatonin supplementation in the drinking water has a systemic effect, affecting several physiological systems. On the other hand, central Mel infusion predominantly affects the CNS. To test if the reducing food intake effect of Mel was centrally mediated, ICV injections, into the third ventricle, of vehicle (ringer), or Mel were performed (Figure 3A). Mel ICV injection reduced food intake in both control and PINX groups, when compared with the respective vehicle-injected groups (group mean ± SEM of 30 daily measurements for each animal; six experimental groups; ****p < 0.0001 vs basal and vehicle).

### Deficiency of Mel Increases the Expression of Orexigenic Neuropeptides in the Hypothalamus, Which Is Prevented by Mel Treatment

Knowing that Mel acts on hypothalamus to reduce food intake, we analyzed the hypothalamic expression of genes related to feeding behavior (Figure 3B) in control, PINX and systemically Mel-treated groups. Concerning the orexigenic peptides, the gene expression of *Npy* was higher in both groups, PINX and PINX + Mel when compared with control group (*p < 0.02). NPY is co-localized with AgRP, another potent orexigenic peptide, and its expression conversely exhibited a significant reduction in the PINX + Mel group (^#^*p* < 0.0001) and an increase in the PINX group (*p < 0.005) when compared with control group. The *Agrp* transcripts reduction in the PINX + Mel group is in agreement with the food intake reduction that we observed in the animals treated with Mel. It is important to note that the magnitude of drop in *Agrp* expression is much greater than the rise of *Npy* transcripts, which may account for the anorexigenic effect of Mel. Contributing to the anorexigenic effect of Mel, the *orexin* transcripts were reduced in the PINX + Mel group compared with PINX (^&^*p* < 0.05). Mel treatment reduced *Pomc* gene expression compared with control and PINX groups (^#^*p* < 0.001). No differences were found in the *Mch* and *Crh* gene expression.

### Mel Treatment Decreases Hyperphagic Response Following Fasting

**Figure 4:**
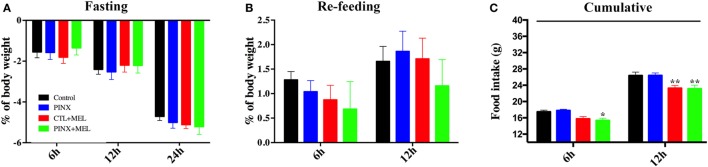
Circadian melatonin treatment effects in the fasting-induced hyperphagia. **(A)** Percentage of body weight loss over the course of 24-h fasting test. Two-way RM ANOVA followed by Tukey post-test. No statistical difference was observed between groups. Interaction *p* = 0.2216 *F*(6, 74) = 1.412; time factor *p* < 0.0001 *F*(2, 74) = 414; groups *p* = 0.9667 *F*(3, 37) = 0.08707; subjects *p* < 0.0001 *F*(37, 74) = 6.708. **(B)** Body weight change throughout the re-feeding period. Two-way RM ANOVA followed by Tukey post-test. No statistical difference was observed between groups. Interaction *p* = 0.3364 *F*(3, 37) = 1.164; time factor *p* < 0.0001 *F*(1, 37) = 31.93; groups *p* = 0.7039 *F*(3, 37) = 4,716; subjects *p* < 0.0001 *F*(37, 37) = 10.32. **(C)** Food intake during the re-feeding period. Data were collected after 6 and 12 h after the food was replaced and shown as cumulative data. Two-way RM ANOVA followed by Tukey post-test, *p < 0.05 vs pinealectomized (PINX) and **p < 0.005 vs control and PINX. Interaction *p* = 0.6128 *F*(3, 37) = 0.6100; time factor *p* < 0.0001 *F*(1, 37) = 373.7; groups *p* < 0.0001 *F*(3, 37) = 10.16; subjects *p* = 0.2340 *F*(37, 37) = 1.272. Data are shown as mean ± SEM, *n* = 10/group.

To understand if the systemic-administered Mel or Mel absence could modulate fasting induced hyperphagia, the animals were tested after 24-h imposed fasting. As seen in Figure 4A, all groups lost weight progressively, reaching a 5% loss after 24 h of fasting and no differences were found between the groups. After 24 h of fasting, the rats were re-fed (Figure 4B). In the first 6 h of food availability, all groups recovered the weight lost during the fasting period and additionally gained body weight after 12 h of re-feeding (Figure 4B). Cumulative food intake was higher in the control and PINX groups compared with the respective Mel-treated groups (Figure 4C; *p < 0.05 vs PINX, **p < 0.005 vs control and PINX), indicating that hyperphagia was higher in the groups that did not receive Mel.

### Acute Leptin Responsiveness Test: PINX Rats Showed No Response to Leptin Injection

**Figure 5:**
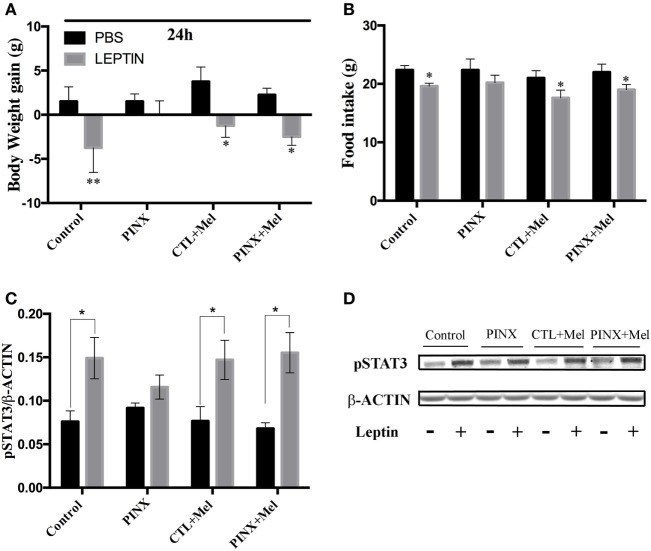
Circadian melatonin absence effects in acute leptin responsiveness test. **(A)** Body weight 24 h after phosphate-buffered saline (PBS) or recombinant leptin injection. *p < 0.01 and **p < 0.0077 vs PBS group. Two-way RM ANOVA followed by Sidak post-test. Interaction *p* = 0.3526 *F*(3, 12) = 1.197; group factor *p* = 0.6264 *F*(3, 12) = 0.6014; treatment *p* = 0.0003 *F*(1, 12) = 26.12; subjects *p* = 0.0440 *F*(12, 12) = 2.791. **(B)** Food intake 24 h after PBS or recombinant leptin injection. *p < 0.05 vs PBS. Two-way RM ANOVA followed by Sidak post-test. Interaction *p* = 0.8775 *F*(3, 16) = 0.2252; group factor *p* = 0.6027 *F*(3, 16) = 0.6359; treatment *p* < 0.0001 *F*(1, 16) = 29.27; subjects *p* = 0.0026 *F*(16, 16) = 4.396. **(C)** Hypothalamic phosphorylation of signal-transducer and activator of transcription 3 (STAT3) 90 min after injection with PBS or leptin, measured by Western Blot. Data were normalized by β-actin amount. *p < 0.05 vs PBS group. Two-way ANOVA followed by Sidak post-test. Interaction *p* = 0.3636 *F*(3, 30) = 1.102; group factor *p* = 0.9571 *F*(3, 30) = 0.1040; treatment *p* < 0.0001 *F*(1, 30) = 25.17. **(D)** Representative immunoblotting of pSTAT3 and β-ACTIN protein content. Data are shown as mean ± SEM, *n* = 5/group.

To unravel possible mechanisms by which Mel affects the energy balance, we assessed the acute anorexigenic response to leptin. Thus, all rats received an intraperitoneal injection of PBS or rat-recombinant leptin 2 h before the dark phase and their food intake and body weight were recorded in the following 24 h. Compared with PBS injection, control, control + Mel, and PINX + Mel groups had a significant reduction in body weight gain. Remarkably, PINX group did not reduce the body weight in response to leptin (Figure 5A, **p < 0.0077 vs PBS and *p < 0.01 vs PBS), suggesting that Mel deficiency causes leptin resistance. The same result was observed in food intake after leptin injection. PINX group did not reduce their food intake in response to leptin (Figure 5B, *p < 0.05 vs PBS). Leptin causes an anorexigenic response *via* activation of leptin receptor signaling pathways in hypothalamic neurons. To further evaluate leptin sensitivity, the capacity of leptin to induce STAT3 phosphorylation in the hypothalamus was analyzed (Figures 5C,D). Leptin evoked STAT3 phosphorylation in control, control + Mel, and PINX + Mel groups (*p < 0.05 vs PBS). In accordance with the food intake and body weight results, leptin failed to induce in STAT3 phosphorylation beyond the observed in PBS-treated rats (*p* = 0.8360).

### PINX Rats Had Lower UCP1 Protein Levels in the BAT and Showed Intolerance to Cold

**Figure 6:**
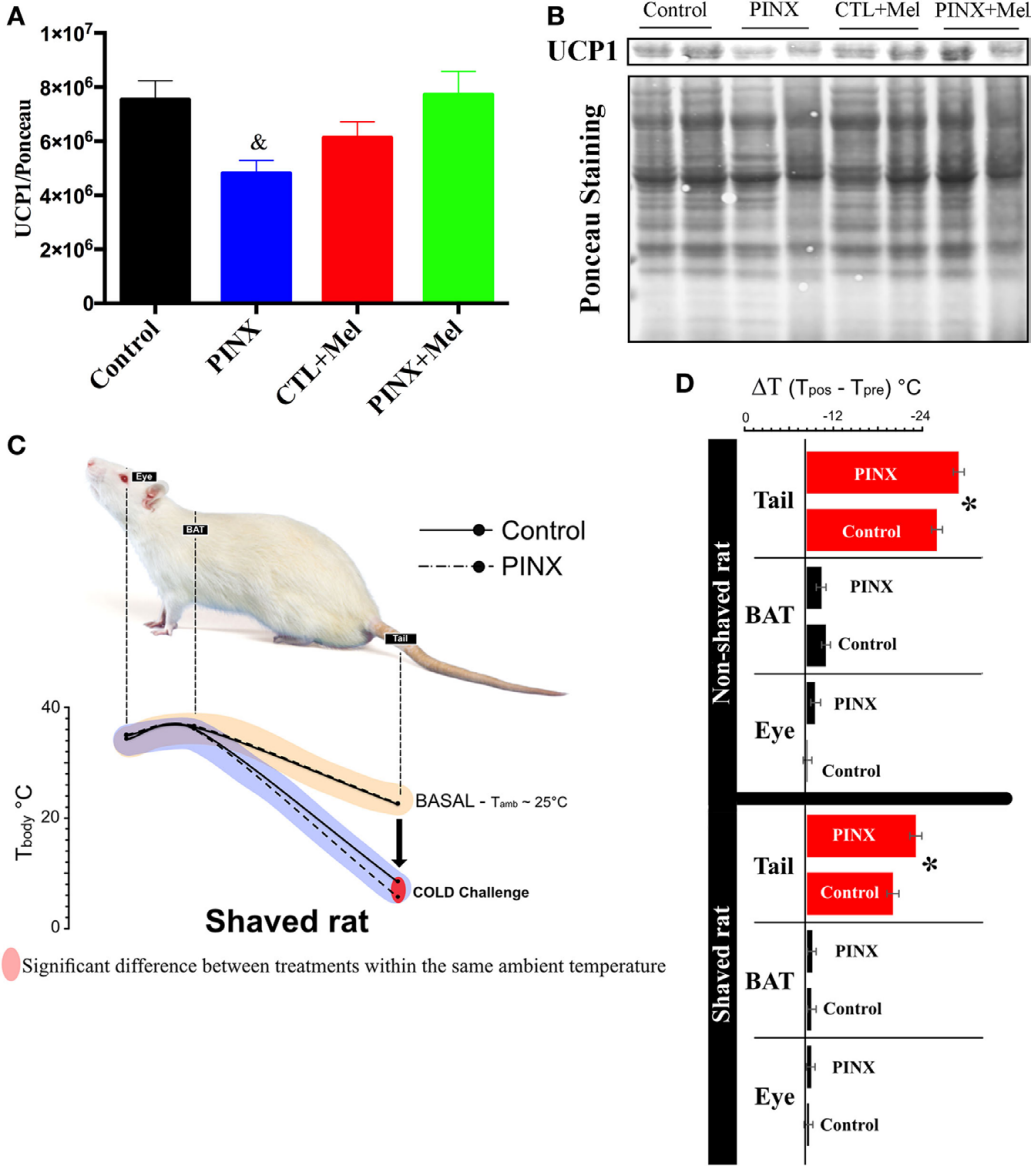
Melatonin (Mel) influence on energy expenditure control. **(A)** Interscapular brown adipose tissue (BAT) content of UCP1 normalized by Ponceau staining. Protein content quantified by Western Blot. One-way ANOVA followed by Tukey post-test, ^&^*p* < 0.05 vs control and pinealectomized (PINX) + Mel. Treatment factor *p* = 0.0191 *F*(3, 25) = 3.979. **(B)** Representative immunoblotting of interscapular BAT content of UCP1 and Ponceau staining. **(C)** Effects of Mel on thermogenic response after cold challenge in unshaved and shaved rat. PINX rats had a more significant drop in tail temperature than the control group *p* < 0.05. **(D)** At the right side, variation of temperatures (delta T_cold_-T_basal_) of eye, BAT and tail in shaved and non-shaved conditions. PINX groups had a greater vasoconstriction in the tails when exposed to the cold in both shaved and non-shaved conditions (shaved: *F* = 34.21, *p* = 0.00004; non-shaved: *F* = 6.16, *p* = 0.0324). One-way ANOVA, followed by Tukey post-test, *p < 0.04 vs control group. Data are shown as mean ± SEM, *n* = 10/group.

The effects of Mel in the energy homeostasis may not be restricted to food intake. To investigate if Mel also modulates biological functions related to energy expenditure, the BAT of control, and PINX rats was studied. We first quantified the levels of UCP1 protein in the BAT by western blot (Figures 6A,B) and Mel absence reduced UCP1 levels compared with control and PINX + Mel groups (^&^*p* < 0.05). To evaluate if the reduction of UCP1 resulted in a lower thermogenic response of BAT, we performed a cold challenge test (Figures 6C,D). We first collected the thermal imaging of eyes, BAT and tail in basal condition (25°C), near what is considered thermal comfort for the animals. No differences were observed in these areas in both groups (Figure 6C, beige-line). Following the cold challenge, the main difference between the groups in thermal imaging was in the tail temperature. PINX rats had a more significant drop in tail temperature than the control group (Figure 6C, purple-line and Figure 6D, *p* < 0.05). In attempt to get a better visualization of BAT, we shaved the dorsal surface of the animals and performed the cold challenge. Again, PINX groups had a greater vasoconstriction in the tails when exposed to cold and no differences were detected in eye or BAT compared with control (Figure 6D, *p* < 0.04).

### PINX Led to Late-Onset Hyperphagia and Overweight

**Figure 7:**
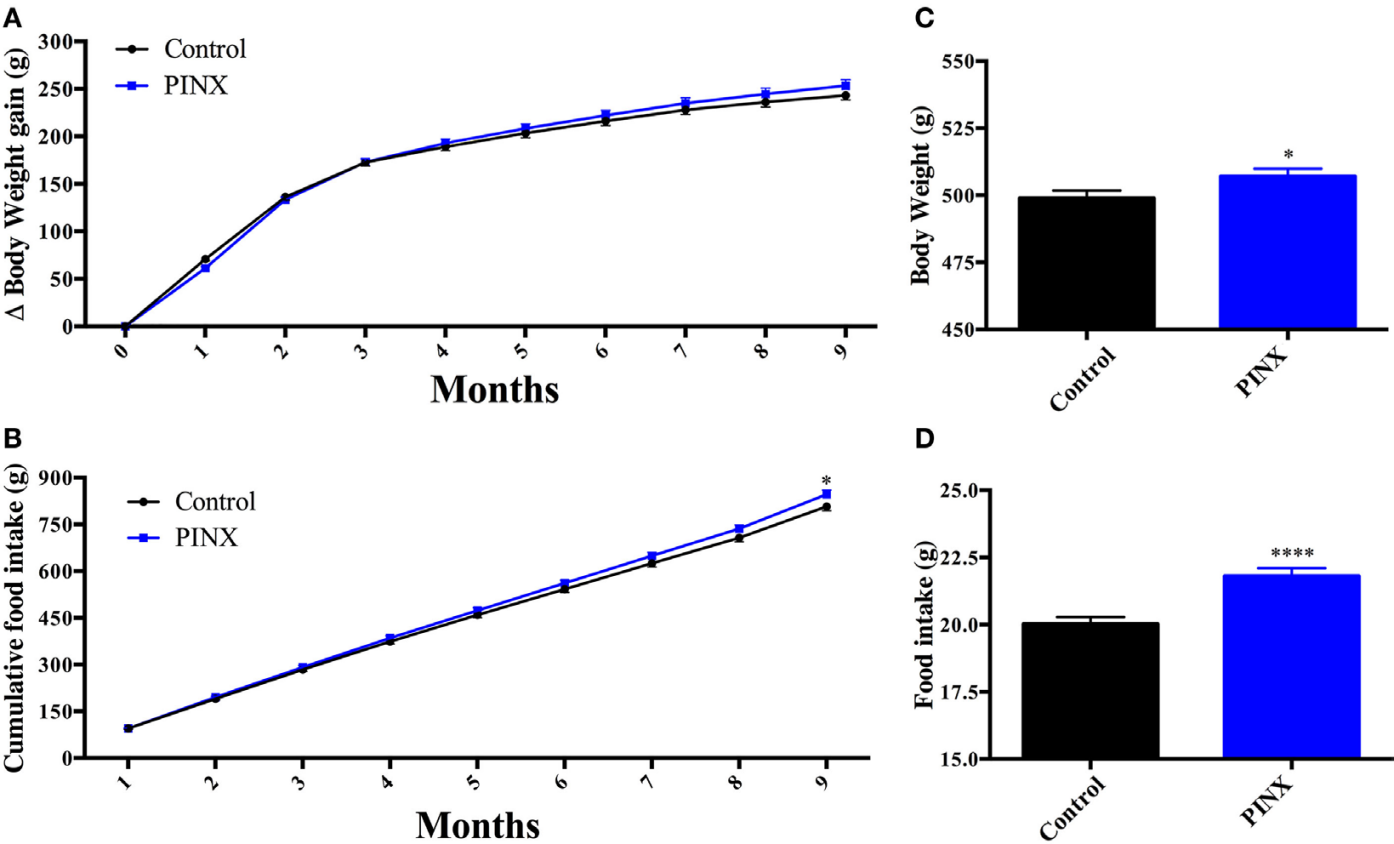
Long-term effects of melatonin absence on rat body weight and food intake. **(A)** Body weight gain over 9 months of pinealectomy surgery. Two-way RM ANOVA followed by Sidak post-test. Interaction *p* = 0.0012 *F*(9, 153) = 3.260; time factor *p* < 0.0001 *F*(9, 153) = 2,461; groups factor *p* = 0.5865 *F*(1, 17) = 0.3075; subjects *p* < 0.0001 *F*(17, 153) = 24.65. **(B)** Cumulative food intake throughout 9 months of pinealectomy. Two-way RM ANOVA followed by Sidak post-test *p < 0.05 CTL vs pinealectomized (PINX). Interaction *p* < 0.0001 *F*(8, 136) = 4.434; time factor *p* < 0.0001 *F*(8, 136) = 6,882; groups factor *p* = 0.1743 *F*(1, 17) = 2.011; subjects *p* < 0.0001 *F*(17, 136) = 35.49. **(C)** Daily body weight at the end of the 9 months protocol. Unpaired *t*-test, *p < 0.05 vs control group. **(D)** Daily food intake at the end of the 9 months protocol. Unpaired *t*-test, ****p < 0.005 vs control group. Data are shown as mean ± SEM, *n* = 10/group.

Initially, we found that PINX animals are centrally resistant to leptin after 13 weeks of pinealectomy (Figure 5C), but they do not present differences in body weight and food intake compared with control group (Figures 1C,D); moreover, they have lower levels of UCP1 (Figure 6A). Consequently, we would expect that they would have a higher body weight and increased food intake compared with controls. In order to determine the long-term metabolic consequences of Mel deficiency, we followed the body weight gain and food intake of PINX and control animals for 9 months (Figure 7). Chronic pinealectomy leads to an increase in food intake (Figures 7B,D, *p < 0.05 and ****p < 0.0001 vs control) and a significant increase in body weight 9 months after pinealectomy surgery (Figures 7A,C, *p < 0.05 vs control). So, taken together these results show that Mel deficiency leads to central leptin resistance in a progressive phenomenon that culminates in increased food intake and body weight.

## Discussion

In the present study, for the first time to our knowledge, we demonstrated that the absence of pineal Mel leads to a progressive leptin resistance. In addition, we demonstrated that the absence of Mel reduced BAT activity, measured by UCP1 levels, alterations in the thermoregulatory mechanisms, increased food intake, resulting in a syndrome that culminates in increased body weight. On the other hand, we show that Mel treatment reduced food intake and body weight, due to its action on the hypothalamus modulating the expression of peptides that are related to food intake.

Some studies have already described the effects of oral Mel supplementation on adiposity reduction and body weight loss in middle-aged rats (9, 10, 32). In contrast to our findings, these reports did not find alterations in food intake, only in feed efficiency. It is important to note that they fed animals with powdered chow (9) what might have led to a different result. In agreement to our work, they found that Mel supplementation reduced plasma leptin levels to the same levels of young animals (9, 32).

The same effect on food intake was demonstrated either when Mel was directly injected in the third ventricle, or orally administered. This result highlights the fact that Mel, in order to modulate food intake, seems to act directly on hypothalamic nuclei and circuits. More recently, MT2 receptor immunoreactivity was moderately detected in the ventral part of the ARH and the medial eminence (33), fundamental areas in the control of food intake that are also target sites for leptin action. Based on these observations, we propose that Mel may modulate feeding behavior by acting directly in the ARH.

An additional means by which Mel may modulate food intake is *via* an action on the hypothalamic suprachiasmatic nucleus (SCN), a region rich in MT1 and MT2 Mel receptors (33). Mel modulation in the SCN is usually associated to its chronobiotic effects, controlling the mechanism of the circadian clock (34–36) acting through MT2. Additionally by acting on MT1, Mel modulates the firing rate of SCN neurons, modulating signal transmission down its projections pathways. Recently, Buijs et al. (37) demonstrated that SCN interaction with the ARH is essential for circadian rhythmicity. They observed that interruption of SCN-ARH communication desynchronizes the ARH causing animals to lose locomotor activity, body temperature, and corticosterone secretion circadian rhythms, without affecting the rhythmicity of the SCN itself. As well documented (6), Mel participates in the circadian organization of energy balance and its association to the circadian activity-feeding—high-energy expenditure/rest-fasting—low-energy expenditure cycle. This system would be an indirect and complementary way of Mel modulating energy metabolism and the SCN, another putative central nucleus where Mel and leptin action might interact, in addition to the ARH (38). However, since our analysis were performed on whole hypothalamus we cannot specify the interaction between SCN and ARH as responsible for the phenotype we observed, further studies on micro dissected nuclei are needed to elucidate better the mechanism underneath.

Melatonin caused a significant reduction in the *Agrp* transcripts and the absence of Mel in the PINX groups led to increased levels of *Agrp* mRNA expression. This result is of particular interest because AgRP neurons are sufficient to orchestrate feeding behavior even under physiological, circadian, and hypothalamic gene expression conditions that are typically associated with satiety. AgRP neurons evoke feeding response independently of suppression of melanocortin pathway (39).

Examination at the global pattern of gene expression of appetite-related peptides in the hypothalamus, we noticed that in the PINX group *Npy* and *Agrp* gene expressions are upregulated and *Pomc* and *Cart* gene expressions show a tendency to be downregulated, which justifies the increase of food intake in the long-term after the pinealectomy. PINX + Mel group also presented upregulated *Npy* gene expression but, in a compensatory mechanism, *Agrp* mRNA expression dropped almost 50% and a downregulation in orexin was also observed. Thus, the balance between orexigenic and anorexigenic peptides in the Mel-treated group results in a negative feeding balance.

Consistent with the present work in mammals, data from other species also showed a similar anorexic effect of Mel (14, 40–42). In zebrafish (*Danio* rerio), Mel significantly reduces food intake and the reduction is in agreement with the changes observed at the molecular level, a significant rise in genes enconding molecules involved in feeding inhibition, such as leptin and MC4R, and a significant reduction in the orexigenic signals including ghrelin and NPY (14). In goldfish (*Carassius auratus*), intraperitoneal injection of Mel or its agonist 2-iodomelatonin significantly decreased food intake (40). Similarly, it has been shown that Mel inhibits feeding behavior in European sea bass (*Dicentrarchus labrax*) in a dose-dependent manner when administered orally in gelatin capsules (41).

To evaluate the hypophagia induced by leptin, food intake, and body weight were assessed after an acute intraperitoneal leptin injection and compared with previous PBS injection. Control, control + Mel, and PINX + Mel showed significantly reduced food intake and body weight while PINX group did not respond to the leptin injection. As previous mentioned, leptin actions are mediated by Jak-Stat3 pathway; under that circumstance, hypothalamic phosphorylation of STAT3 was assessed. Once again, leptin did not evoke phosphorylation of STAT3 in the PINX group, indicating a putative state of leptin resistance. These results indicate that Mel is required to maintain leptin sensitivity, independent of body adiposity. The present work is the first, to our knowledge, to demonstrate that the absence of pineal Mel leads to leptin resistance—the basis of obesity. This finding is very important because previous data show a correlation between several states characterized by reduction in Mel production as, aging, diabetes, shift work, exposure to artificial light at night, with obesity (6, 43). In particular, aging constitutes a complex and dynamic process where, among other features, mechanisms regulating energy balance are changing in comparison with young individuals. At middle age, i.e., 5 months, rats show decreased energy expenditure and responsiveness to anorexigenic hormones such as α-MSH, and these changes develop further until they reach a nadir at 12 months of age (44). In our study, we show that Mel treatment reduces body weight in 5-month-old rats, which suggests interaction of Mel in the melanocortin system and the regulation of body weight with age.

Hyperleptinemia is another feature of leptin resistance. It was shown that pinealectomy resulted in elevated circulating leptin levels, whereas the circadian rhythm of leptin secretion remained conserved (45, 46). Although our results did not show a difference in the circulating leptin concentration between control and PINX groups, they do show that circadian Mel treatment reduced circulating leptin both in intact and PINX rats.

Another important finding in the present study is that PINX rats have lower levels of UCP1 protein in the BAT compared with control. Moreover, Mel replacement therapy in PINX rats is able to restore UCP1 expression in the interscapular BAT to the levels of the intact animals. BAT is involved in non-shivering thermogenesis and due to its high-metabolic activity, is the major determinant of energy expenditure (47, 48). Furthermore, it is known that Mel has a hypertrophic role in BAT (49). Moreover, Teodoro et al. (50) showed that absence of Mel reduces BAT-dependent energy expenditure during the active circadian phase of rats and Mel replacement increases the energy expenditure.

Tan et al. (49) hypothesize that there is an association between Mel synthesis reduction—for example, by night light exposure or aging—and body weight gain. They postulate that if Mel acts recruiting BAT in humans like in small mammals, people exposed to daily long photoperiods should have less functional BAT and may gain more body weight.

The effect of Mel in thermoregulation comes from the discovery of its receptors in specific arteries of rats, including the caudal artery (51), this indicates that Mel may modulate body temperature also through tail-heat loss. Besides that, more recently, it was demonstrated that leptin modulates body temperature by controlling tail-heat loss (52). In the present work, when the animals were cold challenged, PINX animals increased the vasoconstriction in the tail to decrease the tail-heat loss to maintain a proper body temperature. This result points, as expected, to a lower recruitment of BAT in the PINX rats in spite of the present thermographic image does not show the putative difference between the groups.

It should also be noted that leptin actions are partly mediated by the ERK pathway. The anorectic response to leptin can be reversed by blockade of PI3-kinase and ERK. Hypothalamic ERK is a significant downstream target for the effects of leptin to regulate food intake, body weight, and thermogenic sympathetic outflow to BAT. Also, reduced PI3K activity in neurons of the ventromedial nucleus of the hypothalamus leads to a reduction in the thermogenic functions of BAT by suppressing UCP1 expression, indicating PI3K signaling in those neurons is required for mediating acute effects of exogenous leptin on energy homeostasis (53, 54). Mel is known to induce tyrosine phosphorylation of IRS-1/PI3K “*per se*” when injected directly into the lateral ventricle (55). So, considering that the absence of pineal Mel could also impair this alternative pathway, further analysis is necessary to elucidate that mechanism.

Collectively, these results show that the absence of circulating pineal Mel leads to leptin resistance and in a long-term increase of energy intake. At the same time, a reduction of energy expenditure occurs which explains the increasing in body weight of PINX animals. Conversely, when Mel was administrated, an anti-obesogenic effect was observed.

## Ethics Statement

All the experimental procedures were approved by the Ethics Committee on Animal Use of the Institute of Biomedical Sciences of the University of São Paulo, under the number 86/2016.

## Author Contributions

DB and JC-N conceived and designed the experiments. DB, RP, RD, AR-L, RS, GG, JA-S, FA, RM, JD, and LM-T performed the experiments. DB, GG, and JC-N analyzed the data. JC-N provided essential reagents. DB and JC-N wrote the manuscript. JD, RR, and JC-N collaboration in the revision of the submitted manuscript. DB, JD, RR, and JC-N ensuring a proper explanation to possible questions that could be raised regarding accuracy and scientific integrity of the submitted manuscript. All authors read and approved the final version of the manuscript.

## Conflict of Interest Statement

The authors declare that the research was conducted in the absence of any commercial or financial relationships that could be construed as a potential conflict of interest.
